# Thoracolumbar Kyphosis Is a Risk Factor for Proximal Junctional Kyphosis After Isolated Posterior Surgery for Lenke-5C Curvature

**DOI:** 10.3390/jcm14144913

**Published:** 2025-07-10

**Authors:** Nobuki Tanaka, Tetsuro Ohba, Kotaro Oda, Marina Katsu, Hayato Takei, Kai Mizukami, Go Goto, Hirotaka Haro

**Affiliations:** Department of Orthopedic Surgery, University of Yamanashi, Chuo-City 409-3898, Japan; tooba@yamanashi.ac.jp (T.O.); koda@yamanashi.ac.jp (K.O.); marinak@yamanashi.ac.jp (M.K.); takeiha@yamanashi.ac.jp (H.T.); kmizukami@yamanashi.ac.jp (K.M.); ggto@yamanashi.ac.jp (G.G.); haro@yamanashi.ac.jp (H.H.)

**Keywords:** adolescent, kyphosis, lordosis, prevalence, scoliosis

## Abstract

**Background/Objectives**: This study aimed to examine the occurrence and risk factors for proximal junctional kyphosis (PJK) in relation to preoperative sagittal alignment, particularly the shape of thoracolumbar kyphosis (TLK) and the proportion of lumbar lordosis. **Methods**: We recruited 38 consecutive patients with adolescent idiopathic scoliosis (AIS) who underwent isolated posterior fusion. Participants were categorized according to the presence or absence of PJK at 1 year postoperatively (PJK+ or non-PJK) and by preoperative TLK status (positive or negative; pre-TLK+ or pre-TLK, respectively). We compared spinal parameters preoperatively, immediately postoperatively, and at 1 year postoperatively between groups. **Results**: Among the 38 participants, PJK occurred in 21 patients (55.3%). The PJK group had significantly larger preoperative TLK and LDI values and decreased postoperative TLK and LDI. Simple linear regression revealed a moderate positive correlation between ΔPJA and preoperative TLK as well as a fair positive correlation between ΔPJA and changes in TLK and LDI. The prevalence of PJK was high (83.3%) in the pre-TLK+ group (24 patients), and preoperative LDI was significantly larger compared with the pre-TLK group. However, TLK and LDI were significantly decreased after surgery in the pre-TLK+ group. **Conclusions**: Patients with Lenke-5C curvature who exhibit positive preoperative TLK are at a very high risk of developing PJK after isolated posterior surgery. Preoperative sagittal alignment should be considered when planning the extent of sagittal correction.

## 1. Introduction

Scoliosis is considered a three-dimensional deformity. The condition is typically evaluated using the Lenke classification, which is considered to be the gold standard for classifying adolescent idiopathic scoliosis (AIS) deformity and is used to guide surgical planning [1,2].

Assessments based on coronal plane variations have been refined so that surgery can be planned considering factors such as the Lenke classification as well as postoperative correction by selective fusion, last touching vertebra, and S-line [3,4]. In contrast, sagittal plane deformities include thoracic hypokyphosis (TK), increased upper lumbar lordosis (LL), and cervical kyphosis. These variations have been less frequently discussed in the context of surgical planning [5,6]. Nonetheless, this is being increasingly acknowledged, with several recent reports providing new analyses of sagittal plane deformities [7,8,9]. Abelin et al. suggested that surgical planning should incorporate sagittal plane deformity classification, particularly through evaluation of thoracolumbar kyphosis (TLK) and TK [9]. Indeed, it is not uncommon to encounter patients with AIS who share the same Lenke classification but exhibit considerable differences in the sagittal plane, particularly in thoracolumbar shape (lordosis or kyphosis) and the configuration of LL (upper LL or kyphosis).

The surgical goal for AIS is to correct the spinal deformity to achieve harmonious alignment in coronal and sagittal planes. Recent reports focusing on patients with AIS classified as Lenke 5C revealed that proximal junctional kyphosis (PJK) occurs in 18.6–32.5% of cases, which represents a considerable proportion [10,11,12]. While the risk factors for PJK have been well reported, little information is available regarding differences in sagittal alignment among Lenke-5C patients and the mechanisms underlying PJK [10,11,12]. Thus, the present study aimed to analyze postoperative alignment changes and patient outcomes, and to evaluate whether these outcomes were related to preoperative sagittal differences in Lenke-5C patients.

Particularly, we examined the occurrence of PJK at our institution and evaluated risk factors in relation to preoperative sagittal differences; with specific attention to TLK and the proportion of LL.

## 2. Materials and Methods

### 2.1. Study Design

This study was conducted in accordance with the Declaration of Helsinki and approved by the Institutional Review Board of Yamanashi University Hospital (approval No. 2556; 14 April 2018). Written informed consent was obtained from the parents or guardians of all participants.

### 2.2. Study Population

We retrospectively reviewed the medical records of consecutive patients with AIS who underwent posterior spinal isolated fusion surgery from August 2012 to the present. Inclusion criteria were as follows: diagnosis of AIS with major thoracolumbar/lumbar (TL/L) curves (Lenke type 5C), completion of a minimum of 24-month follow-up at our university hospital, aged below 20 years, and selective posterior fusion with all-pedicle screw fixation. Exclusion criteria were included congenital or syndromic scoliosis.

### 2.3. Surgical Procedure

All surgeries were performed by a single spine surgeon (T.O.) using posterior fixation with all-pedicle screws within the TL/L curve, without extending to the major thoracic curve, which is described as isolated TL/L fusion.

### 2.4. Radiographic Measurements

All participants underwent radiological evaluations of the whole spine preoperatively, within 1 month postoperatively, and at the 1-year postoperative follow-up. Radiographic findings include anterior and lateral radiographs of the whole spine in the standing position. In the lateral view, participants stood with knees locked, feet shoulder-width apart, elbows bent, finger placed in the supraclavicular fossa on each side, and looked straight ahead. Cobb angles were measured on the whole-spine radiographs, and the major curves were measured according to Lenke’s established method. The main thoracic (MT) Cobb angle, TL/L Cobb angle, pelvic incidence (PI), pelvic tilt (PT), sacral slope (SS), LL, L4-S angle, TLK, TK, and proximal junctional angle (PJA) were also measured from the same radiographs. The lordosis distribution index (LDI) was calculated as L4-S angle/LL × 100. We defined PJA as the angle between the inferior endplate of the upper instrumented vertebra (UIV) and the superior endplate of UIV + 2. Positive values of TLK and PJA indicated kyphosis, while negative values indicated lordosis. Participants were categorized into PJK and non-PJK groups based on the occurrence of PJK at 1 year postoperatively. Participants were further categorized into the pre-TLK+ or pre-TLK groups according to the preoperative TLK values (positive or negative, respectively).

Radiographic measurements were obtained manually by one author. PJK was defined as increased PJA at least 10° greater. These criteria are widely used in patients with AIS [13,14]. In this study, we defined PJK as PJA increased 10° greater during the 1 year operation from the postoperation referred from past report [15].

We conducted both intra-observer and inter-observer reliability for Pre TLK, Pre LDI, and ΔPJA. Each parameters were measured by two spine certified physicians (N.T and T.O). Correlation coefficients were below (intra-observer error, inter-observer error), Pre TLK (0.9661, 0.9714), Pre LDI (0.9434, 0.9485), and ΔPJA (0.9368, 0.9573). Each observer error reliability was acceptable.

### 2.5. Clinical Outcomes

Health-related quality of life was evaluated preoperatively and at 1 year postoperatively using the Scoliosis Research Society (SRS) outcome tool (SRS-22 version).

### 2.6. Statistical Analysis

Continuous variables are reported as means ± standard error based on three independent measurements. Categorical variables are expressed as percentages. Summary statistics were calculated using Prism (version 8.0; GraphPad Software, La Jolla, CA, USA). The parameters determined by sagittal or coronal radiography were individually compared for validity using simple liner regression (Pearson correlation coefficients). Correlation coefficients of 0.00–0.25, 0.25–0.50, 0.50–0.75, and >0.75 were interpreted as little, fair, moderate-to-good, and excellent relationships, respectively. Statistical significance was accepted at *p* < 0.05. Post-hoc power analyses were performed to estimate effect sample size.

## 3. Results

### 3.1. Study Population and Characteristics

We recruited a total of 38 participants for final analysis. Baseline characteristics and radiographic parameters are presented in Table 1 for the total population and for the PJK and non-PJK groups individually. There were no significant differences between the groups in age, BMI, Risser sign, or number of fixed vertebrae. However, preoperative TLK, L4-S angle, and LDI were significantly greater in the PJK group, with TLK showing a positive value. In terms of postoperative parameters, the PJK group had significantly larger positive TLK and TK values. Comparing postoperative and preoperative values, TLK and L4-S angle were markedly decreased in the PJK group, resulting in significant reductions in LDI. At 1 year postoperatively, LL, L4-S angle, TLK, and TK were significantly increased in the PJK group compared with the non-PJK group. The ΔPJA was also significantly greater in the PJK group compared with the non-PJK group, while SRS-22r scores were significantly lower.

### 3.2. Correlation Between Change in Proximal Junction Angle and Other Parameters

**Figure 1:**
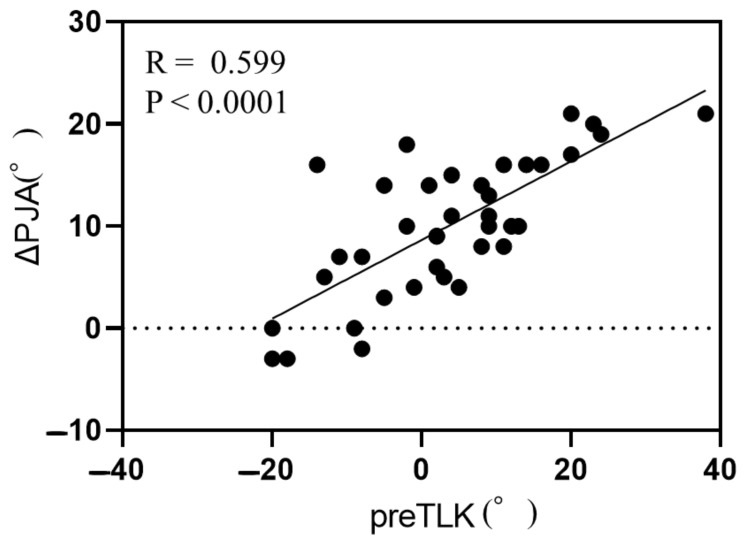
Correlation between change in proximal junction kyphosis and pre thoracolumbar kyphosis. Abbreviations: PJA, proximal junctional angle; TLK, thoracolumbar kyphosis.

**Figure 2:**
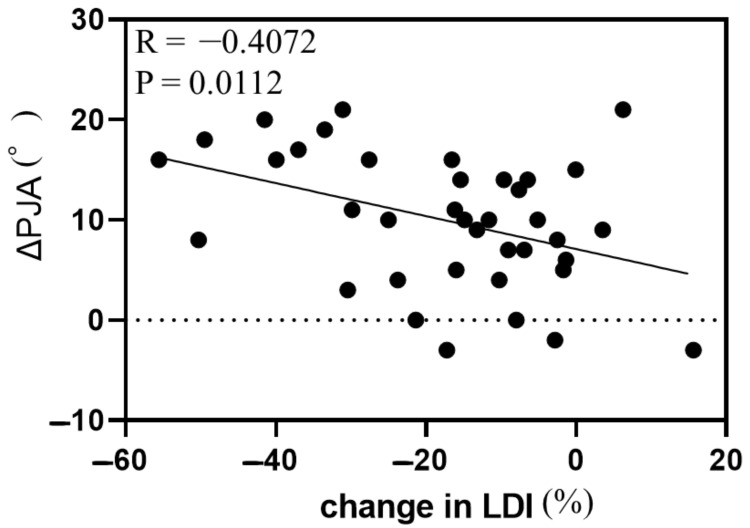
Correlation between change in proximal junctional angle and change in lordosis distribution index. Abbreviations: PJA, proximal junctional angle; TLK, thoracolumbar kyphosis.

**Figure 3:**
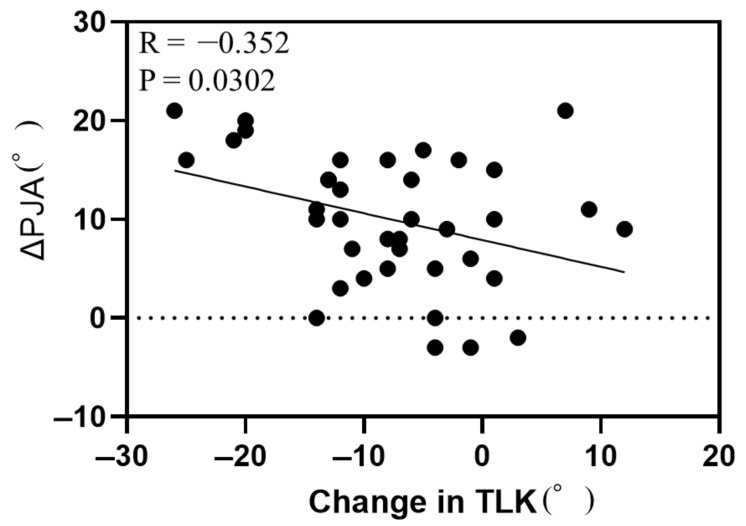
Correlation between change in proximal junctional angle and change in thoracolumbar kyphosis. Abbreviations: PJA, proximal junctional angle; TLK, thoracolumbar kyphosis.

Simple linear regression analysis between ΔPJA and demographic data and radiographic parameters revealed significant correlations between ΔPJA and BMI, preoperative L4-S angle, preoperative TLK, postoperative TLK, postoperative TK, TL/L correction ratio, change in L4-S angle, change in LDI, change in TLK, and 1-year-postoperative parameters (LL, L4-S angle, LDI, TLK, and TK) (Table 2). Of these parameters, preoperative TLK, change in LDI, and change in TLK showed particularly strong correlations with ΔPJA (Figure 1, Figure 2 and Figure 3).

### 3.3. Influence of Preoperative Thoracolumbar Kyphosis

The pre-TLK+ and pre-TLK- groups (24 and 14 patients, respectively) differed significantly in the occurrence of PJK (83.3% vs. 21.4%, respectively; Table 3). There were no significant differences in age, BMI, Risser sign, or number of fixed vertebrae between the groups. In preoperative parameters, TL/L Cobb angle, L4-S angle, LDI, and TLK were significantly larger in the pre-TLK+ group, as was the presence of PJA as a spinal shape. Postoperatively, MT Cobb angle, TLK, and presence of PJA were significantly larger in the pre-TLK+ group, but there were no longer significant differences in L4-S angle or LDI.

Comparing preoperative with postoperative parameters, the pre-TLK+ group exhibited significant decreases in L4-S angle, LDI, and TLK compared with the pre-TLK group. At 1 year postoperatively, PT, LL, L4-S angle, LDI, TLK, TK, and PJA were significantly larger in the pre-TLK+ group compared with pre-TLK-group. ΔPJA was significantly higher in the pre-TLK+ group compared with the pre-TLK group. There was no significant difference in SRS-22r scores between both groups.

### 3.4. Post-Hoc Power Analysis

The observed incidence of PJK was 83.3% (20/24) in the pre-TLK(+) group and 21.4% (3/14) in the pre-TLK(−) group (Cohen’s h = 1.34). With the present sample size (24 vs. 14 patients) and α = 0.05, the achieved power to detect this effect was 0.98. By contrast, the same sample would provide a power of 0.24 to detect a clinically important 20-percentage-point difference (45% vs. 25%) and 0.10 for a 10-percentage-point difference (35% vs. 25%).

### 3.5. Illustrative Cases

**Figure 4:**
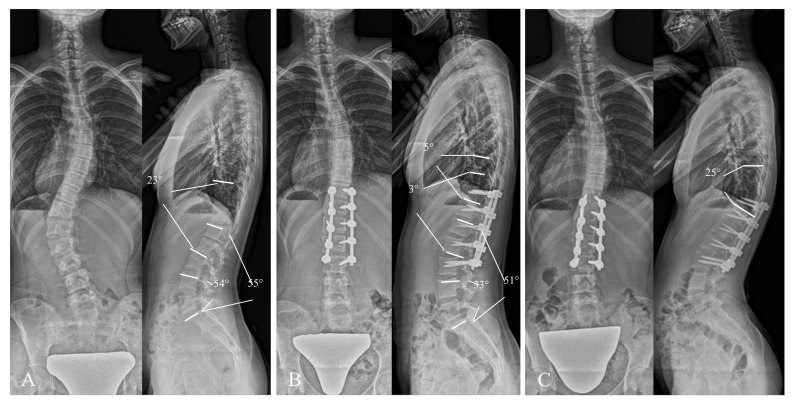
Illustrative radiographs of a patient with positive preoperative thoracolumbar kyphosis who developed proximal junction kyphosis at 1 year postoperatively. (**A**): Preoperative standing whole-spine posteroanterior and lateral radiographs. Thoracolumbar lordosis = 23°, lumbar lordosis = 55°, L4-S angle = 54°, and lordosis distribution angle = 98%. (**B**): Immediately postoperative standing whole-spine posteroanterior and lateral radiographs. Thoracolumbar lordosis = 3°, lumbar lordosis = 51°, L4-S angle = 33°, lordosis distribution index = 65%, and proximal junctional angle = 5°. Thoracolumbar lordosis was changed by −20° and lordosis distribution angle decreased by 33%. (**C**): One-year postoperative standing whole-spine posteroanterior and lateral radiographs showing the occurrence of proximal junctional kyphosis. Proximal junctional angle increased to 25°at 1 year postoperatively (ΔPJA = 20°).

Figure 4 shows a 15-year-old female in the pre-TLK+ group who developed PJK. Preoperative values for TLK and LDI were 23° and 98%, respectively; TLK was corrected to 3°, and LDI decreased to 65% after posterior selective fusion. Postoperatively, PJA was increased by 5°, reaching 25° at 1 year postoperatively (ΔPJA = 20°), accompanied by compensatory increases in LL, LDI, and TK.

**Figure 5:**
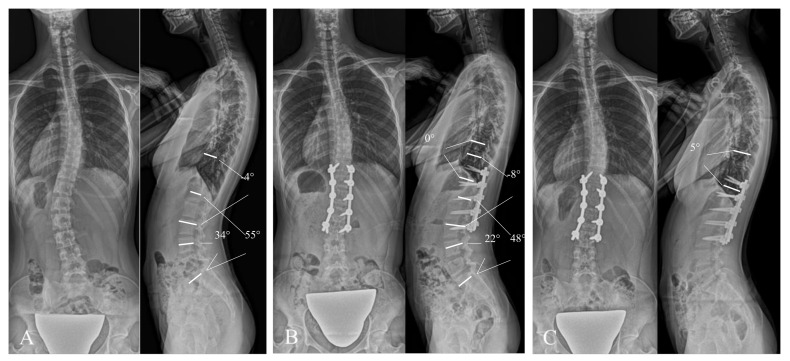
Illustrative radiographs of a patient with negative preoperative thoracolumbar kyphosis who maintained alignment at 1 year postoperatively. (**A**): Preoperative standing whole-spine posteroanterior and lateral radiographs. Thoracolumbar kyphosis = 4°, lumbar lordosis = 55°, L4-S angle = 34°, and lordosis distribution index = 62%. (**B**): Immediate postoperative standing whole-spine posteroanterior and lateral radiographs. Thoracolumbar kyphosis = 8°, lumbar lordosis = 48°, L4-S angle = 22°, lordosis distribution index = 46%, and proximal junctional angle = 0°. Thoracolumbar kyphosis changed by −4°and lordosis distribution index decreased by 16%. (**C**): One-year postoperative standing whole-spine posteroanterior and lateral radiographs showed no evidence of proximal junctional kyphosis. Proximal junctional angle was slightly increased to 5°at 1 year postoperatively (ΔPJA = 5°).

Figure 5 shows the case of a 13-year-old female categorized into the pre-TLK group who maintained alignment. Preoperative TLK and LDI were −4° and 62%, respectively. After posterior selective fusion, TLK increased to 8° and LDI decreased to 46%. A slight increase in PJA was recorded from 0° postoperatively to 5° at 1 year postoperatively (ΔPJA = 5°). Other spinal parameters were maintained until 1 year postoperatively.

## 4. Discussion

A major limitation of this study is its retrospective design and relatively small sample size and only 1-year follow-up. Although the cohort was sufficiently powered to detect the very large risk difference observed between the pre-TLK(+) and pre-TLK(−) groups (effect size h = 1.34, achieved power = 0.98), our post-hoc analysis indicates that the study was underpowered (power < 0.30) to detect more moderate, yet still clinically relevant, effects. For instance, to reliably detect a 20-percentage-point absolute risk increase in PJK—a threshold considered clinically significant—with 80% power at a two-sided α of 0.05, a prospective study would require approximately 90 patients per group. Therefore, while our finding of positive preoperative TLK as a major risk factor is statistically robust, the absence of other significant factors may be due to a type II error. Additional case accumulation is necessary to verify our findings and to identify other potential risk factors with smaller effect sizes.

Recent series suggested that Lenke 5C patients with a rigid or structurally significant compensatory thoracic curve—particularly those showing a preoperative type-C coronal pattern—may benefit from extending the fusion into the thoracic curve [16]. Nevertheless, our investigation intentionally focused on cases treated with isolated thoracolumbar/lumbar fusion to evaluate the outcomes of this strategy in isolation.

The findings presented here indicate that patients with Lenke 5C spinal curves who underwent limited isolated posterior fusion exhibit a surprisingly high incidence of PJK. This is consistent with several previous reports on PJK occurrence and risk factors. For example, in a systematic review of PJK in AIS, Katzouraki et al. found the occurrence of PJK to be 0–46% and reported higher preoperative TK, increased postoperative TK, and increased LL to be risk factors for PJK [17]. Despite the huge number of subjects, their analysis included different Lenke classifications and analyzed isolated and non-isolated fusion together.

Zhou et al. focused on patients with Lenke 5C spinal curvature and reported an 18.6% PJK occurrence after posterior fusion, including non-isolated fusion, and suggested that the main risk factors might differ depending on whether PI is low or high [10]. They recommended avoiding overcorrection of LL and ensuring that the UIV not be at or cephalad to the apex of TK in patients with low PI. Although they did not identify a difference in PJK incidence among patients with Lenke 5C spinal curves in relation to low or high PI, this insight offers a new perspective on PJK pathology [10].

Few studies specifically addressed isolated posterior fusion for patients with Lenke 5C spinal curves. Bai et al. described one of the few studies on this topic, in which they reported the incidence of PJK to be 32.5% following isolated posterior fusion [11]. Large preoperative TK and preoperative PJA accompanied by spontaneous correction of unfused thoracic curves could cause proximal buckling stress, leading to PJK. Intraoperative disruption of posterior structures at the UIV and UIV + 1 located at the TL junctional region was also implicated.

Oba et al. introduced the sagittal S-line tilt (SSLT), defined as the line connecting the anterior end of the cephalad endplate of the UIV and the anterior end of the caudal endplate of the LIV on lateral standing radiographs. They reported the occurrence of PJK to be 21%, and the change in PJA to be moderately positively correlated with preoperative SSLT; thus, SSLT was concluded to be an independent predictor of PJK following isolated posterior fusion for Lenke 5C spinal curves [12]. Notably, SSLT is mainly indicative of thoracolumbar shape (TLK) and upper LL (LDI) in isolated thoracolumbar fusion.

The present study revealed PJK to be more frequent among patients with large positive preoperative TLK values, and thus lower preoperative LDI. Although we found surgical correction to generally change TLK toward the lordotic direction, large positive preoperative TLK values tended to persist, with larger postoperative TLK and large TK as a reciprocal change. Lordotic change led to upper LL and reduced postoperative LDI. Postoperatively increased TK could not be maintained; this resulted in PJK development at 1 year postoperatively. Comparing pre-TLK+ and pre-TLK groups revealed that the pre-TLK group had whole-lumbar gentle lordosis/TLK lordosis and likely hypothoracic kyphosis, while the pre-TLK+ group had large lower LL and kyphotic TLK, and PJA connecting to the upper relatively gentle kyphotic TK. Corrective fusion led to TLK lordotic correction with increased upper LL, decreased LDI, and reciprocally increased TK, which might account for the high occurrence of PJK in the pre-TLK+ group.

The occurrence of PJK associated with UIV selection at the thoracolumbar junctional area in isolated fusion has been reported to be relatively high [18]. In our study, we found that almost all patients experienced postoperative lordotic changes in TLK and decreased LDI following surgery.

Overcorrection of the thoracolumbar curve is a known risk factor for PJK and may increase the load at the UIV level due to the rigid construct [19]. Our data support this, as we observed a fair correlation between the correction ratio and ΔPJA. Flexibility around the thoracolumbar junctional area might be an important consideration when evaluating PJK risk; however, this was beyond the scope of the present study and so was not analyzed. Although we observed significantly lower SRS-22r scores among participants with PJK at 1 year postoperatively, the long-term clinical significance remains unclear, as the numerical difference was only 0.1. Furthermore, this finding conflicts with other reports that found no statistical differences in SRS-22r scores between PJK and non-PJK groups [12,20]. However, Bai et al. reported that preoperative PJK was associated with worse appearance scores at the final follow-up (≥3 years) [11]. Thus, compensatory increases in LL, TLK, and TK might not lead to satisfactory outcomes in middle age or beyond. It is therefore important to consider the potential negative implications of developing PJK during surgical planning and to adapt strategies for patients with Lenke-5C curvature, particularly those with preoperative positive TLK. Abelin et al. classified patients with AIS into four groups according to the sagittal pattern to evaluate TK and positive TLK as well as long thoracolumbar lordosis with cervicothoracic kyphosis [9]. They recommended straightening the TL junction in cases of positive preoperative TLK. In contrast, our findings suggest that correction of TLK in patients with positive preoperative TLK causes decreased LDI, reciprocal increases in TK, and ultimately, the occurrence of PJK.

The risk factors for PJK are multifactorial; therefore, factors such as fusion levels, flexibility, and correction ratio must all be carefully considered to avoid PJK following selective posterior fusion in patients with Lenke-5C curvature. Preoperative positive TLK appears to be a clear risk factor, making surgical planning for these patients particularly challenging. The strategy for isolated posterior fusion for preoperative positive TLK would be avoiding TLK correction. Otherwise, we would chose non-isolated fusion or anterior thoracolumbar fusion to prevent posterior element injury. However, further research is needed to clarify causality and determine the optimal approach to minimize PJK risk. A deeper insight into correction may improve clinical decision-making and, therefore, patient outcomes.

## 5. Conclusions

Patients with Lenke 5C spinal deformity who exhibit positive preoperative TLK are at a very high risk of developing PJK after isolated posterior fusion. Preoperative sagittal alignment should be considered when planning the extent of sagittal correction.

## Figures and Tables

**Table 1:** Study population and characteristics.

	All Patients (*n* = 38)	PJK Group (*n* = 21)	Non-PJK Group (*n* = 17)	*p*
Age at surgery (year)	15.6 ± 2.0	15.3 ± 1.8	15.9 ± 2.2	0.3965
Sex (Male:Female)	0:38			
BMI	20.6 ± 1.4	19.4 ± 1.3	18.6 ± 1.4	0.081
Risser sign	4.5 ± 0.7	4.3 ± 0.7	4.7 ± 0.5	0.0773
Number of fixations	4.3 ± 1.2	4.2 ± 1.3	4.3 ± 1.1	0.9225
UEV				
T9	3 (8%)	1 (4.8%)	2 (11.8%)	
T10	12 (31.6%)	8 (38.1%)	4 (23.5%)	
T11	18 (47.4%)	9 (42.9%)	9 (52.9%)	
T12	5 (13.2%)	3 (14.3%)	2 (11.8%)	
UIV				
T8	1 (2.6%)	1 (4.8%)	0	
T9	3 (7.9%)	0	3 (17.6%)	
T10	9 (23.7%)	8 (38.1%)	1 (5.9%)	
T11	17 (44.7%)	7 (33.3%)	10 (58.8%)	
T12	7 (18.4%)	4 (19%)	3 (17.6%)	
L1	1 (2.6%)	1 (4.8%)	0	
The LEV				
L3	23 (60.5%)	13 (61.9%)	10 (58.8%)	
L4	15 (39.5%)	8 (38.1%)	7 (41.2%)	
LIV				
L2	1 (2.6%)	1 (4.8%)	0	
L3	24 (63.2%)	18 (85.7%)	16 (94.1%)	
L4	3 (7.9%)	2 (9.5%)	1 (5.9%)	
UIV-UEV > 0 (shorter)	13 (34.2%)	8 (38.1%)	6 (35.3%)	
UIV-UEV < 0 (longer)	7 (18.4%)	5 (23.8%)	1 (5.9%)	
LIV-LEV < 0 (shorter)	13 (34.2%)	8 (38.1%)	5 (29.4%)	
LIV-LEV > 0 (longer)	1 (2.6%)	1 (4.8%)	0	
Preoperative angles				
MT Cobb angle (°)	25.3 ± 8.4	26.4 ± 7.8	23.9 ± 9.1	0.3686
TL/L Cobb angle (°)	39.2 ± 8.6	41.2 ± 9.5	36.9 ± 6.9	0.1444
PI (°)	44.5 ± 10.1	42.2 ± 7.0	47.4 ± 12.7	0.1234
PT (°)	12 ± 10.9	12.3 ± 11.9	11.6 ± 9.9	0.8604
SS (°)	32.6 ± 8.9	30.0 ± 9.0	35.8 ± 7.8	0.0434 *
LL (°)	47.8 ± 8.9	48.0 ± 7.9	48.0 ± 10.0	0.8898
L4-S angle (°)	32.7 ± 8.6	35.6 ± 8.7	29.1 ± 7.2	0.0189 *
LDI (%)	69.2 ± 17.3	74.4 ± 15.1	62.7 ± 18.2	0.0358 *
TLK (°)	4.4 ± 12.1	10.7 ± 10.2	−3.2 ± 9.8	0.0001 *
TK (°)	22.1 ± 9.9	23.8 ± 9.3	20.0 ± 10.5	0.2428
PJA (°)	2.2 ± 5.7	3.5 ± 4.8	0.7 ± 6.5	0.1261
Postoperative angles				
MT Cobb angle (°)	16.4 ± 8.0	16.5 ± 8.4	16.4 ± 7.9	0.9491
TL/L Cobb angle (°)	11.2 ± 6.9	10.4 ± 6.3	12.2 ± 7.6	0.4283
PI (°)	44.7 ± 10.0	43.0 ± 7.2	47.2 ± 12.4	0.1553
PT (°)	12.8 ± 9.1	11.9 ± 6.6	13.9 ± 11.6	0.5016
SS (°)	31.8 ± 6.8	30.6 ± 7.2	33.3 ± 6.1	0.2313
LL (°)	46.0 ± 9.7	46.1 ± 9.3	45.8 ± 10.3	0.9209
L4-S angle (°)	23.9 ± 6.7	24.2 ± 6.6	23.5 ± 7.1	0.7321
LDI (%)	51.7 ± 9.5	52.2 ± 9.0	51.2 ± 10.3	0.7341
TLK (°)	−3.2 ± 8.7	0.6 ± 6.8	−7.8 ± 8.7	0.0018 *
TK (°)	28.9 ± 8.7	32.2 ± 6.2	24.8 ± 9.6	0.0063 *
PJA (°)	5.0 ± 5.4	6.1 ± 4.5	3.5 ± 6.2	0.1475
Changes in parameters postoperatively compared with preoperatively				
TL/L correction ratio (%)	71.7 ± 16.3	75.6 ± 12.5	67.0 ± 19.4	0.1055
LL (°)	−1.8 ± 9.4	−1.9 ± 9.4	−1.8 ± 9.7	0.9765
L4-S angle (°)	−8.8 ± 9.4	−11.3 ± 10.3	−5.6 ± 7.4	0.0634
LDI (%)	−17.5 ± 16.6	−22.3 ± 16.7	−11.5 ± 14.8	0.0424 *
TLK (°)	−7.6 ± 8.7	−10.1 ± 9.7	−4.6 ± 6.3	0.0532
TK (°)	6.8 ± 8.4	8.4 ± 8.7	4.8 ± 7.8	0.1843
1-year-postoperative angles				
MT Cobb angle (°)	17.2 ± 7.7	16.0 ± 7.0	18.6 ± 8.4	0.3066
TL/L Cobb angle (°)	14.8 ± 7.6	13.8 ± 6.6	16.1 ± 8.8	0.3626
PI (°)	45.0 ± 9.9	43.0 ± 7.2	48.0 ± 12.0	0.0954
PT (°)	7.6 ± 8.0	6.0 ± 6.7	9.7 ± 9.2	0.1589
SS (°)	37.2 ± 7.3	36.7 ± 7.1	37.9 ± 7.6	0.6147
LL (°)	54.8 ± 9.2	58.4 ± 6.9	50.4 ± 10.0	0.006 *
L4-S angle (°)	31.2 ± 7.1	34.6 ± 5.5	27.1 ± 6.8	0.0006 *
LDI (%)	57.1 ± 10.2	59.4 ± 8.1	54.3 ± 11.9	0.1224
TLK (°)	3.5 ± 13.0	10.1 ± 11.5	−4.7 ± 9.7	0.0002 *
TK (°)	35.4 ± 10.4	41.7 ± 6.5	35.6 ± 8.9	<0.0001 *
PJA (°)	14.9 ± 9.7	21.0 ± 6.9	7.4 ± 8.2	<0.0001 *
ΔPJA (°)	10.0 ± 6.7	14.9 ± 3.7	3.9 ± 4.1	<0.0001 *
Occurrence of PJK	21 (55.3%)			
Scoliosis Research Society-22 scores				
Preoperatively				
Function	2.7 1± 0.3	2.7 ± 0.2	2.8 ± 0.3	0.1862
Pain	2.1 ± 0.3	2.0 ± 0.4	2.1 ± 0.2	0.5512
Self-image	3.2 ± 0.5	3.1 ± 0.5	3.3 ± 0.6	0.5743
Mental health	2.6 ± 0.4	2.6 ± 0.4	2.6 ± 0.4	0.6472
Subtotal	2.6 ± 0.2	2.6 ± 0.2	2.6 ± 0.2	0.6983
1-year postoperatively				
Function	2.7 ± 0.2	2.6 ± 0.4	2.7 ± 0.1	0.0489 *
Pain	2.1 ± 0.5	2.0 ± 0.5	2.2 ± 0.4	0.3946
Self-image	1.9 ± 0.6	2.0 ± 0.7	1.8 ± 0.6	0.505
Mental health	2.7 ± 0.3	2.6 ± 0.3	2.8 ± 0.3	0.1176
Subtotal	2.3 ± 0.2	2.3 ± 0.2	2.4 ± 0.3	0.5065
Satisfaction	2.0 ± 0.8	2.1 ± 0.7	1.9 ± 0.9	0.3959

Interval values represent the mean ± standard deviation. Abbreviations: BMI, body mass index; UIV, upper instrumented vertebra; UEV, upper-end vertebra; LIV, lower-instrumented vertebra; LEV, lower-end vertebra; MT, main thoracic; PI, pelvic incidence; PT, pelvic tilt; SS, sacral slope; LL, lumbar lordosis; LDI, lordosis distribution index (L4-S angle/LL); TLK, thoracolumbar kyphosis; TK, thoracic kyphosis; PJA, proximal junctional angle; ΔPJA, change in PJA at 1 year after surgery; and PJK, proximal junction kyphosis. *p* indicates analysis between PJK group and the non-PJK group. * statistically significant.

**Table 2:** Correlation between change in proximal junction angle and each parameter.

	*p*	r
Age at surgery (year)	0.2358	−0.197
BMI	0.0201 *	0.3546
Risser sign	0.4225	−0.134
Number of fixations	0.6765	−0.06994
Preoperatively		
MT Cobb angle (°)	0.4754	0.1193
TL/L Cobb angle (°)	0.3804	0.1464
PI (°)	0.2354	−0.1972
PT (°)	0.8358	0.03478
SS (°)	0.0967	−0.2734
LL (°)	0.2358	0.197
L4-S angle (°)	0.0018 *	0.4894
LDI (%)	0.0289 *	0.3546
TLK (°)	<0.0001 *	0.599
TK (°)	0.1482	0.2391
PJA (°)	0.0618	0.3059
Postoperatively		
MT Cobb angle (°)	0.8448	0.03284
TL/L Cobb angle (°)	0.0434 *	−0.3294
PI (°)	0.324	−0.1644
PT (°)	0.6632	−0.07298
SS (°)	0.3684	−0.1501
LL (°)	0.546	0.1011
L4-S angle (°)	0.724	0.05921
LDI (%)	0.7062	−0.06321
TLK (°)	0.0022 *	0.4813
TK (°)	0.0204 *	0.3749
PJA (°)	0.0947	0.275
Change (postoperative compared with preoperative values)		
TL/L correction ratio (%)	0.004 *	0.4565
LL (°)	0.6205	−0.08296
L4-S angle (°)	0.0121 *	−0.4032
LDI (%)	0.0112 *	−0.4072
TLK (°)	0.0302 *	−0.352
TK (°)	0.5315	0.1047
1 year operation		
MT Cobb angle (°)	0.1645	−0.2301
TL/L Cobb angle (°)	0.1031	−0.2685
PI (°)	0.2189	−0.2042
PT (°)	0.1628	−0.2311
SS (°)	0.7753	0.04787
LL (°)	0.0002 *	0.5693
L4-S angle (°)	0.0002 *	0.5636
LDI (%)	0.252	0.1905
TLK (°)	<0.0001 *	0.7456
TK (°)	<0.0001 *	0.7429
PJA (°)	<0.0001 *	0.8445

Abbreviations: BMI, body mass index; MT, main thoracis; PI, pelvic incidence; PT, pelvic tilt; SS, sacral slope; LL, lumbar lordosis; LDI, lordosis distribution index (L4-S angle/LL); TLK, thoracolumbar kyphosis; TK, thoracic kyphosis; PJA, proximal junctional angle; and ΔPJA, change in PJA at 1 year postoperatively. * statistically significant.

**Table 3:** Baseline characteristics in relation to preoperative thoracolumbar kyphosis value.

	Pre-TLK + Group *n* = 24 (63.2%)	Pre-TLK Group *n* = 14 (36.8%)	*p*
Occurrence of PJK	83.3%	21.4%	0.0003 *
Age at surgery (year)	15.5 ± 2.2	15.7 ± 1.6	0.7492
BMI	18.9 ± 1.6	19.2 ± 1.2	0.5591
Risser sign	4.6 ± 0.5	4.5 ± 0.7	0.6101
Number of fixations	3.9 ± 0.9	4.5 ± 1.2	0.0728
UEV			
T9	2 (8.3%)	1 (7.1%)	
T10	9 (37.5%)	3 (21.4%)	
T11	11 (45.8%)	7 (50%)	
T12	2 (8.3%)	3 (7.1%)	
UIV			
T8	1 (4.2%)	0	
T9	2 (8.3%)	1 (7.1%)	
T10	9 (37.5%)	0	
T11	7 (29.2%)	10 (71.4%)	
T12	5 (20.8%)	2 (14.3%)	
L1	0	1 (7.1%)	
LEV			
L3	12 (50%)	11 (78.6%)	
L4	12 (50%)	3 (21.4%)	
LIV			
L2	0	1 (7.1%)	
L3	22 (91.7%)	12 (85.7%)	
L4	2 (8.3%)	1 (7.1%)	
UIV-UEV > 0 (shorter)	8 (33.3%)	5 (35.7%)	
UIV-UEV < 0 (longer)	6 (25%)	1 (7.1%)	
LIV-LEV < 0 (shorter)	10 (41.7%)	4 (28.6%)	
LIV-LEV > 0 (longer)	0	1 (7.1%)	
Preoperatively			
MT Cobb angle (°)	27.0 ± 7.8	22.3 ± 8.8	0.0952
TL/L Cobb angle (°)	42.2 ± 7.6	34.1 ± 7.9	0.0034 *
PI (°)	42.2 ± 10.3	48.6 ± 8.8	0.0592
PT (°)	10.0 ± 8.0	15.4 ± 14.3	0.1496
SS (°)	32.1 ± 6.7	33.3 ± 12.0	0.7034
LL (°)	47.3 ± 6.9	48.7 ± 11.9	0.6418
L4-S angle (°)	36.4 ± 7.0	26.3 ± 7.2	0.0001 *
LDI (%)	77.7 ± 13.9	54.5 ± 12.2	<0.0001 *
TLK (°)	12.2 ± 7.0	−8.9 ± 5.2	<0.0001 *
TK (°)	24.0 ± 7.1	18.9 ± 9.2	0.1235
PJA (°)	4.9 ± 4.7	−2.4 ± 4.2	<0.0001 *
Postoperatively			
MT Cobb angle (°)	18.6 ± 7.5	12.8 ± 7.8	0.0299 *
TL/L Cobb angle (°)	12.6 ± 6.3	8.9 ± 7.4	0.1153
PI (°)	42.3 ± 9.8	48.6 ± 9.4	0.0594
PT (°)	11.5 ± 8.0	14.9 ± 10.7	0.2835
SS (°)	31.0 ± 6.8	34.0 ± 6.5	0.1745
LL (°)	45.3 ± 8.4	47.2 ± 11.8	0.5611
L4-S angle (°)	24.6 ± 7.1	22.7 ± 6.2	0.4169
LDI (%)	53.7 ± 10.3	48.3 ± 6.9	0.0895
TLK (°)	1.2 ± 6.4	−10.6 ± 7.0	<0.0001 *
TK (°)	30.8 ± 8.8	25.7 ± 7.6	0.0833
PJA (°)	6.6 ± 5.0	2.1 ± 4.9	0.0101 *
Change post- vs. preoperatively			
TL/L correction ratio (%)	70.5 ± 14.0	73.9 ± 20.1	0.5412
LL (°)	−2.0 ± 9.6	−1.5 ± 9.5	0.8772
L4-S angle (°)	−11.8 ± 10.1	−3.6 ± 5.1	0.0072 *
LDI (%)	−24.0 ± 16.1	−6.2 ± 10.4	0.0007 *
TLK (°)	−11.0 ± 7.5	−1.7 ± 7.5	0.0007 *
TK (°)	6.8 ± 9.3	6.9 ± 6.8	0.9703
1 year postoperatively			
MT Cobb angle (°)	17.8 ± 7.0	16.1 ± 8.8	0.5115
TL/L Cobb angle (°)	15.9 ± 7.4	12.9 ± 7.9	0.2376
PI (°)	42.8 ± 10.1	48.9 ± 8.5	0.0671
PT (°)	5.4 ± 7.5	11.4 ± 7.7	0.0248 *
SS (°)	37.4 ± 7.7	63.9 ± 6.8	0.8224
LL (°)	55.3 ± 7.3	54.0 ± 12.2	0.6934
L4-S angle (°)	33.5 ± 5.5	27.4 ± 8.0	0.008 *
LDI (%)	61.1 ± 9.1	50.4 ± 8.5	0.0011 *
TLK (°)	7.5 ± 9.6	−3.5 ± 15.2	0.0094 *
TK (°)	38.8 ± 9.4	29.6 ± 9.9	0.0075 *
PJA (°)	19.0 ± 6.4	7.9 ± 10.5	0.0002 *
ΔPJA (°)	12.4 ± 5.2	5.8 ± 7.1	0.0021 *

Interval values represent the mean ± standard deviation. Abbreviations: BMI, body mass index; UIV, upper instrumented vertebra; UEV, upper-end vertebra; LIV, lower-instrumented vertebra; LEV, lower-end vertebra; PI, pelvic incidence; PT, pelvic tilt; SS, sacral slope; LL, lumbar lordosis; LDI, lordosis distribution index (L4-S angle/LL); TLK, thoracolumbar kyphosis; TK, thoracic kyphosis; PJA, proximal junctional angle; ΔPJA, change in PJA at 1 year postoperatively; and PJK, proximal junction kyphosis. *p* indicates analysis between the pre-TLK + group and the pre-TLK group. * statistically significant.

## Data Availability

The original contributions presented in this study are included in the article. Further inquiries can be directed to the corresponding author(s).

## Abbreviations

AIS	Adolescent idiopathic scoliosis
LL	Lumbar lordosis
MT	Main thoracic
PI	Pelvic incidence
PJA	Proximal junctional angle
PJK	Proximal junctional kyphosis
PT	Pelvic Tilt
SRS	Scoliosis Research Society
TL	Thoracolumbar
TLK	Thoracolumbar kyphosis
UIV	Upper instrumented vertebra
